# Minimally invasive non-thermal laser technology using laser-induced optical breakdown for skin rejuvenation

**DOI:** 10.1002/jbio.201100083

**Published:** 2011-11-01

**Authors:** Louis Habbema, Rieko Verhagen, Robbert Van Hal, Yan Liu, Babu Varghese

**Affiliations:** 1 Department of Dermatology, Medisch Centrum 't GooiBussum, The Netherlands; 2 Care and Health Applications group, Philips Research EuropeHigh Tech Campus 34, 5656 AE Eindhoven, The Netherlands

**Keywords:** skin rejuvenation, laser-induced optical breakdown, collagen, wrinkle reduction

## Abstract

We describe a novel, minimally invasive laser technology for skin rejuvenation by creating isolated microscopic lesions within tissue below the epidermis using laser induced optical breakdown. Using an in-house built prototype device, tightly focused near-infrared laser pulses are used to create optical breakdown in the dermis while leaving the epidermis intact, resulting in lesions due to cavitation and plasma explosion. This stimulates a healing response and consequently skin remodelling, resulting in skin rejuvenation effects. Analysis of *ex-vivo* and *in-vivo* treated human skin samples successfully demonstrated the safety and effectiveness of the microscopic lesion creation inside the dermis. Treatments led to mild side effects that can be controlled by small optimizations of the optical skin contact and treatment depth within the skin. The histological results from a limited panel test performed on five test volunteers show evidence of microscopic lesion creation and new collagen formation at the sites of the optical breakdown. This potentially introduces a safe, breakthrough treatment procedure for skin rejuvenation without damaging the epidermis with no or little social down-time and with efficacy comparable to conventional fractional ablative techniques. (© 2012 WILEY-VCH Verlag GmbH & Co. KGaA, Weinheim)

## 1. Introduction

Laser- and light-based skin rejuvenation techniques typically balance efficacy, social down-time, and side effects [1–7]. The golden standard for laser resurfacing based on ablative lasers such as CO2 and Er:YAG is associated with severe side effects, significant social down-time and risk of complications [1, 2]. In contrast, non-ablative photothermolysis creates thermal damage in the dermis without causing significant epidermal removal or injury. Even though the side effects associated with these methods are fewer and the social down-time is shorter, the clinical results showed limited efficacy [3, 4]. Ablative fractional resurfacing based on fractional photothermolysis was recently introduced as an intermediate approach to provide efficacy with reduced side effects [5, 6]. In this method, arrays of micro thermal zones are created, leaving the interconnecting tissue intact. The clinical data for ablative fractional resurfacing, which requires application of a local anesthetic, shows a low complication rate with rapid healing times and minimal down-time, especially for singlepass treatments. Therefore, fractional lasers have attracted interest in recent years because of the fewer side effects and the clinical improvement. However, with multiple passes, the ablative damage accumulates, which increases thermal damage and healing time [7].

In general, all these techniques rely on selective photothermolysis based on linear absorption of optical energy by the skin's constituents. In order to influence the ratio of damage between the dermis and epidermis the optical energy is sometimes focused at the desired depth, and/or the epidermis is cooled superficially. This field has been studied intensively over the last decades, but no revolutionary approach has been identified that is capable of accurately defining the balance between efficacy, safety, social down-time and pain perception.

In this manuscript, we demonstrate a novel, minimally invasive laser technology for skin rejuvenation that creates microscopic damage to areas within tissue below epidermis level using laser-induced optical breakdown (LIOB) [8–16]. This leads to a healing response and consequently skin remodeling, resulting in skin rejuvenation effects. A prototype device has been constructed and used in this study. To obtain proof-of-principle for the purpose of skin rejuvenation by means of LIOB, we present a histological timeline showing evidence of microscopic lesion creation and new collagen formation at the sites of the optical breakdown in the days following the treatment on *in-vivo* human skin. The potential benefit of this technology is that it provides an effective and safe procedure without damaging the epidermis.

## 2. Materials and methods

We have developed in-house a prototype device for the validation of the new concept of skin rejuvenation using laser-induced optical breakdown (LIOB). The device has obtained a declaration of European Conformity (CE) by Philips Electronics N.V. and was reviewed and approved by the Medical Ethics Committee (Medisch Ethische Toetsings Commissie, METC). The prototype device consists of a base station, an articulated arm, a treatment hand piece and a computer. The base station houses an optical system, a cooling system and electronics. The optical system comprises a pulsed laser source, beam shaping optics and mirrors to guide the laser beam to the hand piece *via* the articulated arm. The laser source is a flash lamp pumped SLM TEM00 Nd:YAG laser which delivers sub-nanosecond light pulses of 1064 nm with pulse energies in excess of 0.15 mJ at focus level inside the skin, which is sufficient to cause optical breakdown. Even though this wavelength is absorbed by water and melanin within the skin, the amount of absorbed and scattered light is rather small. Blood is a strong absorber at this wavelength, but because the volume fraction of blood is a few percent at this depth, the average absorption coefficient that affects light penetration is low. The hand piece consists of a focusing system that focuses the laser beam to a focal spot (Φ < 10 μm) within the skin, sufficient to cause optical breakdown, resulting in a cavitation bubble in the skin. The cavitation bubble increases the area of damage and the lesion size ultimately reaches 0.1 to 0.2 mm in diameter [9]. During treatment, an optical matching liquid is applied to effectively couple the light in to the skin. We have applied few drops of Phosphate Buffered Saline (PBS) solution on the skin using a pipette. The effective numerical aperture inside the skin is 0.68. The optical scanner integrated inside the hand piece is able to treat a selected area of the skin (19×19 mm^2^) at a scanning speed up to 5 mm/s with a focus depth which is adjustable in the range of 100 to 750 mm below the surface of the skin. The scan pattern is controlled by an algorithm implemented in a computer. The pitch of the grid of laser lesions inside the skin can be adjusted in the range of a few micrometers up to several millimeters. A glass plate is used to protect the optical elements from direct contact with the treated skin surface and to define the depth of treatment in the skin.

To verify whether the proposed technique is capable of creating sub-epidermal lesions while leaving the epidermis intact, a series of ex-vivo experiments on human skin samples have been performed. Fresh human skin samples of Fitzpatrick skin type II–III were obtained from a local hospital under supervision of the METC. The full thickness skin samples were soaked in Phosphate Buffered Saline (PBS) solution and stored and transported in a refrigerator. Prior to the actual treatment, samples of about 20×20 mm^2^ with a thickness of about 1 mm were prepared.

To evaluate the effectiveness and safety for skin rejuvenation, an *in-vivo* study was performed on the buttock area of five paid human volunteers, age 20 to 65 years, with Fitzpatrick skin types II and III, in accordance with medical ethical committee in Catharina- Ziekenhuis in Eindhoven, the Netherlands. The targeted lesion depth was 190 mm, and four different density coverages were tested: 2.5, 5, 10 and 20 percent, expressed in terms of the average lesion crosssectional area immediately post treatment as a fraction of the non-lesion skin surface area. Histological investigations were performed four times: immediately (∼30 min), 3 days, 7 days, 30 days and 180 days after treatment. This requires 4 or 5 skin biopsies taken from the treated area, including one control skin biopsy from an untreated area that is adjacent to the treated zone. This required 4 to 5 skin biopsies taken from treated area including one control skin biopsy from an untreated area that is adjacent to the treated zone. The formation of new collagen was investigated by immuno-histological analysis or other relevant histological analyses. To assess the long-term skin responses, histological analyses were performed 6 months after the treatment.

The type and severity of post-treatment skin responses, such as pigmentary changes, pin bleeding and any other side effects were noted and documented for evaluating whether the severity of the observed side effects were acceptable by the test subjects. Photographs taken before and at various time intervals after treatment were also analyzed. The sensations perceived by the test subjects during the treatment session were assessed by means of a questionnaire for evaluating the subjective experience of the treatment.

## 3. Results

**Figure 1:**
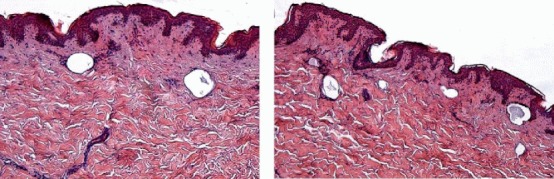
*Ex-vivo* skin specimen treated with the new prototype device, stained with haematoxylin and eosin (H&E) demonstrating the creation of lesions inside dermis of human skin.

The *ex-vivo* skin specimen treated with the prototype device, stained with haematoxylin and eosin is shown in Figure 1. The figure shows that the LIOBinduced microscopic lesion patterns occur at dermal level only, whereas the top layers of skin, i.e., stratum corneum and epidermis, are fully intact. The lesion pattern observed in the skin specimen is consistent with the pattern of treatment. Furthermore, the tissue between the microscopic lesions appears unaffected. This demonstrates the primary proof-ofprinciple that it is a safe and effective procedure for creating microscopic isolated damage to areas within tissue in the dermis without damaging the epidermis. This leads to a healing response and consequently skin remodeling, resulting in skin rejuvenation effects.

**Figure 2:**
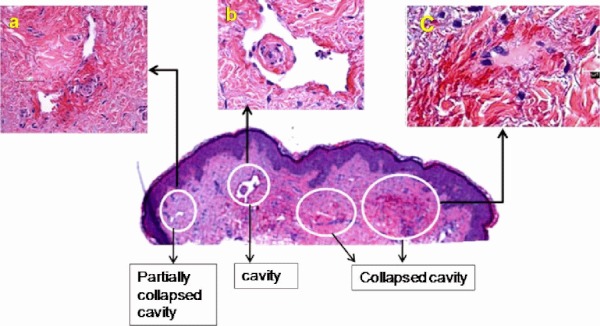
Specimen from Subject 5, 30 minutes after treatment stained with H&E. Lesions are characterized by cavity, partially collapsed cavity, or collapsed cavity. Insets depict the features of partially collapsed cavity (a); cavity (b) and collapsed cavity (c), where large amount of erythrocytes accumulated in the damaged zones are clearly visualized.

**Figure 3:**
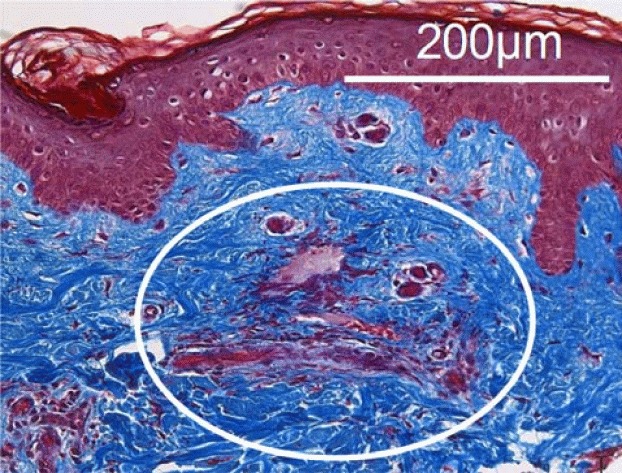
Specimen from subject 1, 30 minutes after the irradiation, stained with Masson Trichrome. Damage is identified by a lesion accompanied with large amount of erythrocytes (circled zone). Damage occurs around 200 mm below skin surface while the epidermis and stratum corneum remain unaffected.

In the histological analysis of the samples taken from the panel test with live subjects, a pattern of microscopic lesion creation was clearly depicted in all subjects, which is consistent with the pattern of LIOB treatment. Figure 2 shows the histologically stained specimen with haematoxylin and eosin (H&E) taken 30 minutes after the treatment. The micro-lesions were observed in the dermis, while the top layers of skin, i.e., the stratum corneum and epidermis, are unaffected. The depth of the micro-lesions is around 200 mm, which is consistent with the device settings of 190 mm. In principle, cavities within the skin dermal level should be generated after light exposure. However, we do not observe cavities at all treatment sites. We attribute this to a collapsing phenomenon: when micro-damages were created by the treatment, skin may disrupt around the damaged sites, leading to (partial) collapse of the cavities, which makes the cavities less visible in the H&E stained skin specimen. To overcome this, another staining technology, the Masson Trichrome stain, was performed. As shown in Figure 3, the micro-lesion is more distinguishable by staining with Masson Trichrome, in which proteins are stained in blue and cells are stained in dark brown.

**Figure 4:**
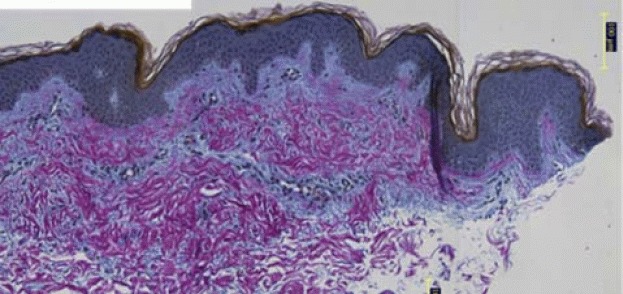
Skin specimen taken at 30 days after treatment was stained with Herovici staining. The mature collagen (collagen I) stained in red whereas the young collagen (collagen III) stained in blue.

To assess the formation of new collagen formation, Herovici staining was performed. The Herovici staining technique is able to distinguish between young and mature collagens by differentially staining mature collagen (collagen type I) in red and young collagen (collagen type III) in blue. As shown in Figure 4, the papillary layer of skin contains more fine and young collagen than the deeper reticular layer in the dermis. Micro-lesions from the skin sample taken immediately after the treatment were again visible in Herovici staining. The appearance of the new collagen corresponded to the depth of the created lesions and was absent in control biopsies. No obvious young collagen formation was observed from the skin specimens taken at 3 days and 7 days after the treatment. This is expected, as new collagen takes several days to form. The strands of new collagen formation, an indicator of wound healing, were detected in the skin specimens taken 30 days after the treatment (Figure 4), illustrating the progress of skin rejuvenation after treatment. No obvious new collagen formation has been found in skin specimens 180 days after the treatment, possibly implying the completion of skin rejuvenation.

The assessment of post-treatment skin response revealed that the treatment was safe: no pre-described side effects were observed in the periods: shortly, 3 days and one week after receiving the treatment. The micro-lesions in the skin at 3 days and 7 days after the treatment were not as visible as those from skin specimens taken immediately after the treatment, but macrophages, whose role was to phagocytose cellular debris, were noticeably accumulated in some sites which were believed to be associated to the positions of the micro-lesions inside the dermis.

In all cases, treatment led to erythema that was noticeable immediately after treatment and was no longer visible 30 minutes after treatment. Edema was observed in all cases a few minutes after the treatment and resolved in about 60 minutes. Erythema and edema were defined as “end-points” of the proposed study. The appearance of both implied that the treatment was effective and a skin healing response was expected to occur. During the calibration session, petechiae – tiny intra-dermal bleeding – were observed in all five test subjects after the exposure while no pin-bleeding was observed. Petechiae cleared up between 7 to 14 days. Petechiae severity increased with increasing coverage and depended on treatment depth. No pigmentary changes, bleeding, blistering, scarring or other side effects were observed in the treated area during the post-treatment follow-ups. Patient ratings of treatment discomfort ranged from not perceptible (40 percent) to slightly painful (40 percent) at worst, with the remarkable observation that most subjects rated higher coverage densities as more acceptable.

## 4. Discussion

In this paper we have presented a novel minimally invasive non-thermal laser technology using laser induced optical breakdown (LIOB) for skin rejuvenation. The physical principle of the method presented is fundamentally different from the laserbased methods and devices that are commonly used for skin rejuvenation. The existing laser devices, which are based on selective photothermolysis, rely on linear absorption of optical energy by the skin's constituents. In contrast, LIOB is a non-linear absorption processes which can occur only when the irradiance is sufficiently high to produce a critical free-electron density of about 1021 cm^3^ [11]. Optical breakdown leads to plasma generation by multiphoton and avalanche ionization, followed by explosive vaporization and mechanical expansion in the dermis [13, 14]. The highly confined energy leads to much localized mechanical effects in the form of a microexplosion in the dermis [10, 15]. Laser-induced plasma formation in ocular and aqueous media has been extensively investigated in the past decades and is used in medicine, spectroscopic evaluation of environmental contaminants and optical limiting [10, 16].

Initial *ex-vivo* and *in-vivo* studies performed for evaluating this proposed minimally invasive treatment technique show that the epidermis remains intact while microscopic intradermal lesions are successfully created, which leads to the formation of new collagen. No open wounds were created by the treatment. No pigmentary changes, scarring or other side effects occurred in the course of the study. Subjects did not experience any unpleasant sensation during and post treatment. In other words, the treatment is very acceptable to all test subjects. No anesthesia, cooling plate or other topical applications were needed prior to, during, or after the treatment. The appearance of petechiae during the treatment prolonged the so-called social down-time and was therefore defined as a new side effect. Though neither deemed painful nor associated with any unpleasant sensation, there is a strong need to diminish the occurrence of petechiae. By keeping coverage density maximum at 10 percent and with adjustments of the optical coupling of the device to the skin and treatment depth, problems with petechiae have been largely avoided in a further testing program performed on volar forearm skin. With high coverage density of lesions, there is possibility for damaging the nerves. However, the probability of occurrences is expected to be not higher than the existing ablative and non-ablative techniques. These results indicate that the new prototype device is potentially effective and safe for skin treatment.

The ablative collagen remodeling technique that we have described avoids all damage to the epidermal layers since micro explosions are highly confined to a very small volume in the treated areas. This allows for a larger proportion of the dermal collagen to be treated for high efficacy, without the risk of severe side effects. This will overcome the limitation of present professional skin rejuvenation methods which are lacking in a good balance between safety and efficacy.

This technique enables a breakthrough skin rejuvenation method by introducing a safe treatment procedure without damaging the epidermis, and with no or little social down-time. Furthermore, because the new technique generates lesions at dermal level where the formation of new collagen will occur, we consider that the efficacy of the new technique will be comparable to conventional fractional ablative techniques. The level of safety is the same as with non-ablative techniques because it generates microlesions in the dermis while the epidermis is unaffected. The potential benefit of this technology is that it is a safe procedure with high efficacy and without damaging the epidermis.

## 5. Conclusion

We have developed a novel minimally invasive laser technology based on the principle of laser-induced optical breakdown (LIOB) for skin rejuvenation. The optical breakdown caused by tightly focused near-infrared laser pulses creates a grid of intradermal lesions that lead to skin rejuvenation without affecting the epidermis. Using a prototype device, we have successfully demonstrated the safety and effectiveness of the method for laser skin rejuvenation. Analysis of *ex-vivo* and *in-vivo* treated human skin samples show evidence that the lesions are created only in the treated areas in the dermis. Treatments lead to mild side effects that were controlled by small optimizations of the optical contact and treatment depth within the skin. The perception of the treatment was found to be acceptable for the majority of the test panel, without the use of topicals or systematic anesthesia. This potentially introduces a safe, breakthrough treatment procedure for skin rejuvenation without damaging the epidermis with no or little social down-time and with efficacy comparable to conventional fractional ablative techniques. Further *in-vivo* studies were performed with this device in a pilot panel test to investigate the efficacy of wrinkle and fine line reduction. These results will be reported separately in the near future.
